# Maternal and early life exposures and their potential to influence development of the microbiome

**DOI:** 10.1186/s13073-021-01005-7

**Published:** 2022-01-11

**Authors:** Erin E. Bolte, David Moorshead, Kjersti M. Aagaard

**Affiliations:** 1 Translational Biology and Molecular Medicine Graduate Program, Baylor College of Medicine, 1 Baylor Plaza, Houston, TX 77030 USA; 2 Immunology & Microbiology Graduate Program, Baylor College of Medicine, Houston, USA; 3 Medical Scientist Training Program, Baylor College of Medicine, Houston, USA; 4 Department of Obstetrics and Gynecology, Division of Maternal-Fetal Medicine, Baylor College of Medicine and Texas Children’s Hospital, Houston, USA; 5 Department of Molecular & Human Genetics, Baylor College of Medicine, Houston, USA; 6 Department of Molecular & Cell Biology, Baylor College of Medicine, Houston, USA; 7 Department of Molecular Physiology & Biophysics, Baylor College of Medicine, Houston, USA

**Keywords:** Microbiome, Maternal, Pregnancy, Fetus, Offspring, Cesarean delivery, Gnotobiotic, Germ-free, Placenta, Intrauterine environment

## Abstract

At the dawn of the twentieth century, the medical care of mothers and children was largely relegated to family members and informally trained birth attendants. As the industrial era progressed, early and key public health observations among women and children linked the persistence of adverse health outcomes to poverty and poor nutrition. In the time hence, numerous studies connecting genetics (“nature”) to public health and epidemiologic data on the role of the environment (“nurture”) have yielded insights into the importance of early life exposures in relation to the occurrence of common diseases, such as diabetes, allergic and atopic disease, cardiovascular disease, and obesity. As a result of these parallel efforts in science, medicine, and public health, the developing brain, immune system, and metabolic physiology are now recognized as being particularly vulnerable to poor nutrition and stressful environments from the start of pregnancy to 3 years of age. In particular, compelling evidence arising from a diverse array of studies across mammalian lineages suggest that modifications to our metagenome and/or microbiome occur following certain environmental exposures during pregnancy and lactation, which in turn render risk of childhood and adult diseases. In this review, we will consider the evidence suggesting that development of the offspring microbiome may be vulnerable to maternal exposures, including an analysis of the data regarding the presence or absence of a low-biomass intrauterine microbiome.

Advances in culture-dependent and culture-independent molecular methodologies continue to reveal details of the human-microbe relationship, allowing for increasing identification of “who is there” (microbiota) and what the community is “capable of doing” (functional microbiome). Two working definitions of “microbiome” exist, which collectively frame an emphasis on community functionality. The first definition provided by *Nature* defines “microbiome” as “all of the genetic material within a microbiota (the entire collection of microorganisms in a specific niche, such as the human gut). This can also be referred to as the metagenome of the microbiota.” (https://www.nature.com/subjects/microbiome) The second definition put forward by Whipps *et al.* defines “microbiome” as “a characteristic microbial community occupying a reasonably well defined habitat which has distinct physio-chemical properties. The term thus not only refers to the microorganisms involved but also encompasses their theatre of activity” [1]. Although distinct, these two definitions share the commonality of emphasizing the community’s functional capacity and resultant activity, which can collectively be thought of as the microbial community functionality. Of note, the functionality need not be limited to the metabolic functions of the microbiota *per se.* Rather, there may be functional importance tied to niche occupancy (so-called colonization resistance). This might result from host-independent functions, such as dependence on limited substrates or production of macromolecules which exclude occupancy of other microbes. It may also result from host-dependent functions, such as antigenic exposure resulting in immune tolerance or immune activation. Of note, neither definition requires long-term establishment or high-biomass colonization. In the case of human microbiomes, this functionality may be unique to specific life stages (i.e., child to adolescent, or adult to elder adult), normal physiologic adaptations (i.e., during pregnancy and lactation), or may have a wide dynamic range which oscillates predictably within an individual but widely across the population (i.e., vaginal microbiome community dominance patterns, also called “community state types”). Throughout this review, we presume the understanding that “microbiome” infers a microbial community of live microbes with functionality, recognizing that functionality is not limited to microbial metabolic activity *per se.*

Much effort has been concentrated on demonstrating the association between human health and the functional high-biomass gut microbiome, with evident associations between the microbiome and human neurobehavior [2], metabolism [3], and immunity [4–6]. Clearly, a functional metagenome is critical for normal human physiology and neurodevelopment at all life stages. However, particular attention has been paid to the early life microbiome. “Critical developmental windows” have been identified, during which microbes and their functional pathways are necessary to promote normal metabolic, immune, and neural development [7–10]. However, the origin, timing, and route of the human microbiome acquisition, initiation, integration, and maturation during these “critical developmental windows” remains unresolved.

In this review, our goal is to provide a presentation of the evidence of a relationship between several different maternal exposures, the maternal microbiome, and the offspring’s microbiome and health outcomes. We will explore what has been learned about both the impact and lack of impact with specific maternal exposures on the development of the offspring microbiome. We will review the literature and strength of evidence both supporting and refuting the presence and functional importance of low-biomass intrauterine microbial communities in early development. In particular, we will discuss the placental microbiome and the role it may or may not play in contributing to perinatal outcomes, the offspring’s microbiome or microbiome fitness, and subsequent childhood health and disease. We will also explore the recent published evidence refuting the notion of low-biomass perinatal communities, including intrauterine communities like the placenta. Finally, we will conclude this review with our viewpoint, which challenges the long-held notion that the intrauterine low-biomass community is of little or no functional consequence [11–16]. Rather, we will argue that functional consequence remains to be determined experimentally and with an evidential basis. Solving the mystery of how and when during early development we come to tolerate commensal microorganisms is fundamental and crucial to engaging in safe and efficacious studies and trials which attempt to manipulate our microbiome [17].

Several pregnancy exposures have been associated with alterations of the offspring microbiome, which precede later onset of disease in childhood and adult life. The combination of “nature” and “nurture” has been used over the years to explain the variability in human health, including both the range of susceptibility and severity of clinical disease from one individual to the next, or among different populations. For example, following World War II, undernourished women were observed to have increased risk of miscarriage, birth malformations, and low-birthweight infants [18, 19]. Kermack *et al.* showed that early life exposures lead to specific and predictable effects throughout the life of offspring [20]. More formal evidence continued to accrue following a series of sentinel observations by Barker and his colleagues, which collectively supported the theory that maternal pre-conception and pregnancy exposures can result in long-term health impacts on her offspring [21–27]. Now formally termed the Developmental Origins of Health and Disease (DOHaD) hypothesis, we appreciate today that fetal exposures persistently alter human physiology and behavior in ways that last well into adult life [28]. We and others have described the effect of common pre-pregnancy and pregnancy exposures. We have extensively studied the effect of maternal high-fat diet consumption during pre-conception, pregnancy, and lactation and inhalation/ingestion of polycyclic aromatic hydrocarbons (found in tobacco smoke and petroleum byproducts) during pregnancy on the development of obesity, modulation of immunity, and dysfunction of metabolism in offspring via long-lasting perturbations at the level of the placenta, liver, pancreas, skeletal muscle, thyroid, and digestive tract [29–78]. The mechanisms driving these long-term effects are partially explained by the developing epigenetic code [29–37, 39–55, 59, 60, 63–69, 73, 74, 76] and microbiome [56, 61, 62, 70–72, 77–81]. Specifically, the gut microbiome can contribute small molecules and nutrients to the maternal circulation that can be actively or passively transferred across the placenta (in some, but not all cases) during fetal development [82, 83], leading to varied effects in the offspring. Rodent, primate, and human studies support the observation that certain and key pregnancy exposures can lead to alterations in the maternal and/or offspring microbiomes and offspring health [57, 84–103].

In the subsequent section, we will discuss eight examples of how the offspring microbiome and health are shaped by common maternal exposures: (example 1) changes in the maternal microbiome, (example 2) women who are pregnant and living with a furry pet, (example 3) maternal exposure to herbicides, (example 4) environmental pollutants, (examples 5–7) maternal diet, and potentially (example 8) maternal stress.

## Example 1

It has been established that a wide range of factors may impact the structure and function of the gut microbiome, including but not limited to sex, geography, diet, environment, genetics, and medication. However, what effect does a change in the maternal microbiome (be it transient or permanent) have on the developing fetus or its eventual microbiome? Gomez de Agüero *et al.* addressed this question in a rodent model by transiently colonizing pregnant germ-free mice with a specially engineered *Escherichia coli* strain [103]. This strain of *E. coli* was genetically designed to perish within the intestine; once introduced through oral gavage to the dams, its residence within the intestine was short-lived. Even though active colonization did not occur, the offspring of the *E. coli*-exposed dams displayed enhanced innate immune system development and intestinal epithelial maturity compared to offspring without that maternal exposure. These findings translated to the *E. coli*-exposed offspring having improved intestinal inflammatory response and host-microbial symbiosis to post-natal microbes due to the maternal-microbe interaction.

## Example 2

The environment (both in the home and outside of the home) includes inhalation, absorption, and ingestion exposures, including exposures to domesticated animals (i.e., pets and domesticated farm animals or livestock). It has been demonstrated that children reared with domesticated animals from infancy have reduced prevalence of allergic-related illnesses [104]. Could the effect of such domesticated animal exposures extend prior to birth? Tapiainen *et al.* investigated the effect of common maternal exposures on the human first-pass meconium microbiome [93]. They analyzed delivery mode, perinatal antibiotics, probiotic consumption, and the number of “furry pets” living in the home with the woman during pregnancy. Multivariate analysis indicated that having “furry pets” at home impacted numerous measures of the meconium microbiome: number of operational taxonomic units, Shannon diversity, and relative abundance of Firmicutes, Bacteroidetes, *Staphylococcus* spp., and *Faecalibaterium* spp. Based on the variables that the investigators were able to control for in their analyses, the measures of the microbiome were impacted by “furry pets” in the home independently of delivery mode, perinatal antibiotics, or probiotic consumption in this cohort [93].

## Example 3

The majority of crops consumed in the USA are exposed to herbicides containing chemicals such as glyphosate [105]. These environmental chemicals have demonstrable efficacy and low toxicity; however, some concerns have been raised about the effect of these herbicidal components during pregnancy. In a mouse model of maternal exposure to high-dose glyphosate during pregnancy, juvenile offspring (post-natal day 28) display abnormal behaviors, an altered gut microbiome, and increased levels of acetate in stool [102]. Although it is unlikely that humans are exposed to high-dose glyphosate through consumption of herbicide-treated crops, these data provide one example of how a prenatal environmental chemical exposure can influence the offspring microbiome composition and later gut and behavioral health down the road.

## Example 4

Other ambient (inhalation) and water/soil (ingestion) environmental chemicals like polycyclic aromatic hydrocarbons (PAHs) represent a more common maternal exposure. These noxious organic substances are byproducts of carbon combustion (i.e., vehicle emissions, petroleum processing) and cigarette smoking, and they are associated with contamination of air and soil/aquatic life and perinatal complications. The adverse pregnancy outcomes associated with PAH exposure include, but are not limited to, preterm delivery, low birthweight, neonatal bronchopulmonary dysplasia, child-onset asthma, low cognitive assessment scores in children, neurodevelopmental delay, DNA adduct formation, alteration of DNA methylation patterns, and persistent fetal reprogramming [106–118]. PAHs are also involved in bidirectional effects on maternal physiology, as PAHs can modulate an individual’s microbiome community membership and its function, but the microbiome itself contributes to polycyclic aromatic hydrocarbon transformation and metabolism [119]. Ingested polycyclic aromatic hydrocarbons are capable of disrupting gut microbial enzymes, leading to a state of induced dysbiosis within the gut community [119]. But the relationship is not one-sided. Human commensal microbes can metabolize primary polycyclic aromatic hydrocarbon products (that are inhaled or ingested) and secondary polycyclic aromatic hydrocarbon substances (that are conjugated by the liver) [119]. For example, benzo[a]pyrene (BaP) is a polycyclic aromatic hydrocarbon compound that cannot activate human estrogen receptor; however, the human gut microbiome transforms BaP into an estrogenic compound that can activate human estrogen receptor [120]. While neither the short- nor long-term implications of exogenous estrogen receptor activation in a developing fetus are well-established [121], this is a subject of ongoing research for our laboratory and others.

## Example 5

Maternal diet is an important focus of every pregnancy, leading to decades of landmark studies investigating multiple aspects and components of dietary intake in the context of perinatal (pregnancy and lactation) and offspring outcomes. A very common feature of the maternal diet during pregnancy includes efforts to reduce sugar and/or carbohydrate intake, inclusive of the use of artificial non-nutritive sweeteners. This may occur in the context of a gravidae being diagnosed with gestational diabetes, or with efforts to manage gestational weight gain. Outside of pregnancy, it has been shown that artificial non-nutritive sweeteners alter the microbiome [122] and negatively affect several measures of metabolic health [123–125]. Stichelen *et al.* investigated the effect of non-nutritive sweetener consumption during pregnancy on health outcomes in murine pups [96]. Not only were profound metabolic and hepatic detoxification changes observed, but the pups also exhibited a gut microbiome profile associated with human metabolic disease and obesity (increased Firmicutes and decreased *Akkermansia muciniphila*) at post-natal day 19. Further experimentation revealed that both control and sweetener-fed dams transmit *A. muciniphila* to their newborn pups. Interestingly, over the first 3 weeks of life, pups exposed to maternal sweetener consumption lose this beneficial microbe and acquire metabolic, hepatic, and microbiome structure disturbances.

## Example 6

Another feature of maternal diet during pregnancy may be the woman’s efforts to increase fiber intake, both to avoid the constipation that accompanies physiologic adaptive smooth muscle relaxation and to optimize caloric intake. Pregnant mice fed a high-fiber diet (compared to regular-fiber and no-fiber) produce offspring that are more resistant to allergic disease [88]. Specifically, the maternal high-fiber exposed offspring are resistant to allergic airway disease (a model for human asthma) and produce fewer eosinophils and proinflammatory cytokines at 3 weeks, 6–8 weeks, and 16 weeks old. The protective effects of maternal high-fiber diet exposure were similar in maternal acetate-exposed offspring, where the dams were fed acetate in their drinking water rather than a high-fiber diet. The study’s authors propose that, since increased acetate is observed in serum and feces following high-fiber consumption, acetate may represent the causal mechanism for the anti-inflammatory effects seen in association with maternal high-fiber diet exposure [88]. Other mechanisms may be in play, since protection against atopic disease is also observed with maternal consumption of prebiotic oligosaccharides [84].

## Example 7

Another dietary modification observed in gravidae is the relative increase in fat intake during pregnancy. Studies of the effect of maternal high-fat diet consumption on offspring health have been conducted in multiple animal models. For example, in non-human primates exposed to maternal high-fat diet consumption during pregnancy and lactation and then weaned onto a control diet for at least 6 months, the gut microbiome of offspring is persistently altered compared to their peers who were not exposed to maternal high-fat diet [57]. The maternal high-fat diet exposure is associated with decreases in *Campylobacter* spp. and *Helicobacter* spp. and increases in *Ruminococcus* spp. and *Dialister* spp., which occur independent of juvenile obesity, refractory to probiotic/prebiotic therapy, and observed at least 6 months and up to 2-plus years after the cessation of maternal high-fat diet consumption [57, 77].

## Example 8

Exposure to “stress” during pregnancy is a common concern for many women who are pregnant, as stress can alter biological pathways, including the microbiome [90]. However, prenatal “stress” is a commonly used but poorly defined term, generally associated with both psychological and/or physical symptomology. From a pragmatic perspective, in daily life humans and animals encounter situations that demand adaptation. Stress occurs if adaptation can only be met with great difficulty or is impossible. Because it is both situational and individualized, “stress” is challenging to define and measure precisely [126]. Some studies have used quantitative measures of stress derived from numerical scores generated from standardized, self-reported questionnaires focusing on negative maternal affect, depression, anxiety, worry, perceived stress, and life satisfaction [127–129]. Other studies define stress by major life events [130] or with biological biomarkers like salivary cortisol and alpha amylase [129]. Although the data are divided, some studies of maternal stress during pregnancy report offspring sleep disruption [127], atopic dermatitis [128], lower cognitive development scores [129, 130], and increases in both infectious and noninfectious illnesses [131]. In a rodent model of stress during pregnancy, Jašarević *et al.* [90] demonstrate that maternal stress impacts the post-natal colonic microbiome beginning at post-natal day 2 and extending to post-natal day 28 (latest time point tested). A particularly disrupted microbiome structure was observed in male offspring at post-natal day 28. Interestingly, the changes observed in these males were characteristic of female-typical microbiome patterns, indicating an axis between stress, hormones, and the gut microbiome.

From these eight examples, we learn that in every mammal examined to date, a number of maternal exposures impact the offspring’s health in early life. These functional impacts are often accompanied by alterations in the microbiome community composition and function, with some intervention studies inferring causality. However, it is less clear whether there is a true causal relationship between maternal exposure-driven perturbations in the infant microbiome and the onset of disease in childhood, nor its parlance to adult disease. As one example, data from the Canadian Healthy Infant Longitudinal Development (CHILD) study demonstrated an association between the transient absence of certain gut microbes (Micrococcaceae and Veillonellaceae, genera *Lachnospira*, *Veillonella*, *Faecalibacterium*, and *Rothia*) in the infant stool microbiome at 3 months of life with a subsequent risk of a higher Asthma Predictive Index at 3 years of age [133]. However, those same gut perturbations were not observed in the interval 1 year of age cohort suggesting that the dysbiosis observed at 3 months was transient [132]. These same investigators inoculated germ-free mice with these same four bacterial taxa and observed ameliorated airway inflammation in their adult progeny [132]. However, the nature of this inflammatory suppression was not entirely consistent with the histopathology anticipated with suppression of asthmatic disease and the study was limited by its small sample size [132].

Collectively, these studies and others suggest that perinatal exposures can lead to both short-term and long-term alteration of the offspring’s gut microbes and/or health, and some of these alterations are refractory to post-natal or post-weaning correction. In addition, an emerging body of data have specifically addressed the role of the maternal microbiome during pregnancy and its impact on development of the offspring immune system [9, 52, 103, 134, 135]. However, much of this work remains correlative and not causal, and further data is necessary to understand the molecular mechanisms driving these associations. To this end, in order to further explore examples of association needing causal mechanistic links, in this next section we will review data arising from two examples commonly cited as circumstantial evidence against a role for prenatal exposures having an impact on either short- or long-term alterations to the composition or function of the offspring microbiome. These examples are (1) observations among offspring delivered via Cesarean, and (2) phenotypic analysis of gnotobiotic animal models.

## Example 1: Cesarean delivery and the offspring microbiome—innocent bystander or causal driver?

In 1985, the World Health Organization called for safe delivery practices for women and stated the “ideal” Cesarean delivery rate in a population should be 10–15% [136]. Since that time, the prevalence of Cesarean delivery has increased around the world (i.e., the rate in USA is approximately 31–32% in 2016–2019 [137]). Several renowned leaders focused on maternal and neonatal safe delivery have called for a reconsideration of the WHO and other entities recommendations pertaining to “ideal Cesarean delivery rates” with more precise use of definitions and classifications of the underlying driving factors and indications for Cesarean delivery to arrive at an evidence-based recommendation of what the “right” rate of Cesarean delivery ought to be [138]. In the meantime, a number of researchers are concerned about what effect Cesarean delivery may or may not have on the offspring’s short- and long-term health and microbiome.

Numerous studies report a correlation between Cesarean delivery and long-lasting health risks in term Cesarean-born offspring, including three large cross-sectional and longitudinal studies [139–141]. Specifically, groups reported increased risk of childhood obesity, atopy/asthma, lower cognitive development, and Celiac disease in Cesarean-born offspring [139–149]. Zhang *et al.* performed a meta-analysis of 61 studies to assess the effects of four different delivery modes: elective versus emergent Cesarean delivery and assisted versus unassisted vaginal delivery [139]. They observed increased odds for development of autism spectrum disorder (ASD) and attention deficit hyperactivity disorder (ADHD) in offspring delivered by either Cesarean delivery mode compared to vaginal delivery. However, the authors note they were unable to account for maternal and/or infant confounders due to absent information in the included studies.

Other research groups have utilized regression analysis to include maternal and infant factors alongside Cesarean delivery as variables in offspring health prediction models. These groups did not observe robust long-term negative outcomes in offspring born by Cesarean delivery alone when accounting for maternal socioeconomic characteristics [150], geolocation [151], BMI [150, 152], parity [150], age [150, 152], smoking during pregnancy [150–152], education [151, 152], ethnicity [152], marital status [152], preeclampsia [152], infant sex [151, 152], gestational age (at delivery) [151, 152], and birth weight [151]. Fundamentally, the subject of Cesarean delivery can be described as follows: does the surgical procedure of Cesarean delivery itself impact health outcomes of the offspring, or do other maternal and/or infant covariates (e.g., the “company” that Cesarean delivery keeps) contribute? This question is not simple to address in an experimental model due to the wide range of variables associated with the decision to perform Cesarean delivery, which is likely responsible for the disparate conclusions observed in correlative research studies examining the effect of Cesarean delivery on offspring health. However, it is critical that this research question be sufficiently answered so that obstetricians and pediatricians can understand what exact variables are responsible for (versus covariables associated with) the increased risk of negative health outcomes in Cesarean-delivered children.

If the Cesarean surgery *itself* is causing these adverse short- and long-term outcomes, what would be the underlying molecular mechanism driving this association? Some have suggested that altered first microbial inoculations at birth not including vaginal microbes might be one such mechanistic culprit [153, 154]. In other words, as awareness for the importance of the microbiome and its functional metagenome in human health and development continues to grow, concerns have been raised that failure to expose the emerging neonate to the “proper” community of microbes will lead to adverse clinical outcomes throughout the offspring’s life. Dominguez-Bello and colleagues were the first to study the neonatal microbiome across body sites immediately following delivery in a small case-control study [153]. Across all body sites, vaginally born neonates harbor mostly maternal vaginal microbes, such as *Lactobacillus*, *Prevotella*, or *Sneathia* spp., whereas Cesarean-born neonates harbor mostly maternal skin microbes, such as *Staphylococcus*, *Corynebacterium*, and *Propionibacterium* spp. Subsequent experiments by this group indicate that vaginal seeding of the neonate following Cesarean delivery enriches signature vaginal taxa in the oral, skin, and gut microbiomes of 30-day-old infants [155]. Shao and colleagues further confirm the association between Cesarean delivery and an altered neonatal microbiome composition in a cohort of almost 600 term babies with repeated sampling through the first month (days 4, 7, 21) of life, where disrupted Bacteroides spp. and increased *Enterococcus*, *Enterobacter*, and *Klebsiella* spp. correlated with Cesarean delivery, intrapartum antibiotic use, and lack of breastfeeding [156]. Mueller *et al.* confirmed lower proportions of Bacteroides, *Parabacteroides*, and *Clostridium* spp. in the neonatal gut microbiome of Cesarean-delivered offspring [157]. Many additional studies have similarly reported a correlation between Cesarean delivery and altered microbiome composition profiles in the neonate (see Table 1 of the review by Moya-Pérez *et al.* [200] and review of colonization rates by Shaterian *et al.* [201]) but several questions remain. Do these changes persist? What is the biological, functional consequence of changes in the neonatal microbiome? Are these changes a result of the Cesarean delivery *per se* or with maternal and/or infant variables that accompany and likely drive the need for a Cesarean delivery?

Some groups have identified differences in the Cesarean-born gut microbiome beyond the neonatal period [157, 202–206]. Niu *et al*. [203] queried a cohort of 729 children under 3-years-old and discovered an association with decreased Bacteroidetes in Cesarean-born offspring. Azad *et al*. [207] and Coker *et al*. [208] analyzed term infants repeatedly up to 12 months and observed persistent changes in emergent Cesarean delivered offspring at 12 months old; however, these changes were found to be associated with the intrapartum antibiotic exposure and ameliorated by breastfeeding. These data indicate that covariables of Cesarean delivery play an important role in the microbiome changes associated with Cesarean delivery. In our own study of this issue, multivariate analysis of metagenomes from multiple body sites in our prospective, longitudinal cohort of n=81 maternal-infant dyads enabled us to parse characteristics of the neonatal (obtained at birth) and infant (4-6 weeks of age) microbiome community structure and its function [72]. Consistent with the findings of Dominguez-Bello *et al*. described above, we too observed that among unlabored Cesarean-born neonates, the newborn microbiota and its functional pathways were most similar to the maternal skin and relatively homogenous across all body sites, except meconium [72]. However, the neonatal microbiome from labored Cesarean deliveries more closely resembled the distribution seen in vaginally delivered neonates where the infant microbiota were more similar to the communities of the maternal vagina and skin [72]. From an obstetrician’s perspective, the underlying clinical indication for a scheduled, unlabored Cesarean would be anticipated to significantly differ from that of an intrapartum, labored Cesarean delivery. Overall, variations in microbial community structure of the oral gingiva, nares, and skin (*R*^2^=0.038) by delivery mode were observed immediately following delivery but not by 4 to 6 weeks of age, and no significant differences were ever observed in the meconium or infant stool microbiota by mode of delivery, nor any body site’s functional metabolic pathway [72]. Several antepartum, intrapartum, and/or postnatal exposures were indicated as covariates contributing to microbiome functional pathway differences in generalized linear modeling [72]. Specifically, we observed that maternal high-fat diet, intrapartum antibiotics, and any formula feeding appeared to have the greatest effect on pathway variation in the infant stool, while gestational age and pre-pregnancy BMI had little effect [72]. Of particular interest, given co-linearity with antibiotic use and exclusive breastfeeding practices, Cesarean delivery *per se* did not bear a differential effect on the metagenome nor its function when subject to robust linear mixed modeling controlling for covariates as fixed effects [72]. In other words, there was no discernable impact attributable to Cesarean mode of delivery on the presence or absence of specific microbes comprising the infant microbiome by 4-6 weeks [72]. These findings are further consistent with a cohort comprised of preterm born infants, were there was no observed difference in the gut microbiome community structure by 5 weeks of age by virtue of being born via vaginal or Cesarean delivery [209].

Most of these studies have analyzed the effect of Cesarean delivery on the neonatal and infant microbiome by 16S ribosomal RNA (rRNA)-based detection [153, 155, 157, 202, 203, 205, 207, 210]. 16S amplicon-based sequencing is fast, affordable, and allows taxonomic resolution to the genus level with the aid of algorithms like DADA2 [211]. However, 16S sequencing cannot reliably yield species/strain-level calls. Therefore, 16S sequencing can identify broad taxonomical differences, but is not the optimal tool to identify the functional components of the microbiome. Why should the functional microbiome matter, if we know the taxonomical structure? It is important to remember that microbial nomenclature is not synonymous with microbial functionality. Different microbial strains within a species can contain dissimilar genes and functional capacity (i.e., *E. coli*), and different species can contain functional overlap. For this reason, functional profiling of the microbiome is a necessary next step in understanding the microbiome in any setting [212–214], including that of the Cesarean-delivered offspring. Whole-genome shotgun sequencing (WGS) allows for species and strain-level calls that can specify what functions a given community has or lacks [215, 216]. A few studies have analyzed the effect of Cesarean delivery on the neonatal and infant microbiome by WGS sequencing [72, 156, 204, 206, 210], but only two [72, 210] analyzed the effect of Cesarean delivery on the functional microbiome and saw no lasting impact.

In order to consolidate the discordant conclusions of the effect of Cesarean delivery on the offspring gut microbiome and health trajectories, the causal versus correlative effect of delivery mode must be carefully examined for confounders and company of Cesarean delivery. Such variables include the underlying medical or obstetrical indication or pathology leading to the surgery, antibiotic exposure surrounding delivery, environmental exposure to the neonatal intensive care unit, human milk versus formula feeding, other maternal comorbidities, and yet-unidentified factors. For example, it has been suggested that Cesarean delivery may be associated with altered bacteria-bacteriophage interactions [217]. Bacteriophages (the viruses that infect bacteria) represent a largely unexplored component of the microbiome that may play a role in early development of the infant microbiome. As we recently commented [218], identifying intrauterine transmission of bacteriophages prior to delivery is an exciting new avenue of research. Perhaps bacteriophages represent one of multiple unexplored variables with the potential for intrauterine transmission that may play a role in affecting the gut microbiome of offspring that are born via Cesarean. The maternal dietary and medical conditions which may modulate bacteriophage transmission remain unexplored [218].

In summary, the neonatal microbiome following delivery is different in vaginal versus Cesarean-born neonates, and Cesarean delivery has been associated with a number of offspring health risks [139–141, 153–157, 200–207, 210]. Because early life is a critical window for normal human development, it stands to be concerned about these changes to the neonatal microbiome and potential lasting health impacts during early developmental windows [219].

However, outstanding speculation remains regarding the root cause of the altered neonatal microbiome and offspring health impacts associated with Cesarean delivery [72, 150–152, 207–210]. Namely, it is important to discern the effect of Cesarean delivery itself from the clinical confounders that accompany Cesarean delivery [72, 150–152, 207–210]. Resolution of this discrepancy requires incorporation of the clinical confounders that accompany Cesarean delivery with large, prospective studies naïve to eventual delivery mode. Cesarean delivery is a common and safe abdominal surgery. Ready availability to medically indicated Cesarean with surgically competent providers is crucial in the reduction of maternal and neonatal mortality and decreasing social disparities worldwide (https://www.who.int/reproductivehealth/publications/maternal_perinatal_health/cs-statement/en/). Greater understanding of the effect of birth by Cesarean in-and-of-itself on offspring health is needed without inadvertently diminishing a safe and necessary procedure. These data provoke consideration of the implications that follow if delivery mode does not meaningfully impair the long-term structure or function of the microbiome. One such implication is this: if the moment of delivery does not direct the body’s future interactions with microbes, then when does the developing host receive these instructions? What can the DOHaD hypothesis contribute to this issue? To further contemplate this latter question, we turn to observations arising from functional studies in gnotobiotic animal models.

## Example 2:

## Are gnotobiotic animals “normal”?

One of the most powerful tools available to study host-microbial interactions is the development of germ-free (GF) animal models, originally conceived by Louis Pasteur in 1885 and first cultivated in guinea pigs [220], rats [221], then mice [222]. Gnotobiotic animals are created and reared in environments “free” of (detectable) live bacteria. Their food, water, bedding, and any supplies are autoclaved or filter-sterilized, and sterility is continuously monitored by culture and molecular methods. While GF animals are viable and reproduce despite the lack of live microbes, they are by no means normal. Compared to conventionally colonized animals, GF animals display morphological differences, biochemical abnormalities, atypical neurobehavior, and pronounced immunological changes. GF animals are characterized by prolonged diestrus, small lymph nodes and spleen, thin intestinal villi and lamina propria, increased food/water intake, higher oxidation-reduction potential, altered mucosal enzyme patterns, and decreases in circulating leukocytes, immunoglobulin levels, Peyer’s patch size, intraepithelial T cells, inflammatory response, blood volume, regional blood flow, cardiac output, basal metabolic rate, motor activity, response to catecholamines, body fat, organ sizes, vitamin biosynthesis, enteric bile acid transformation, and intestinal-specific parameters (mass and surface area, peristalsis, epithelial cell renewal, pH levels) [223–234]. It is important to note, though, that in spite of GF and germ-depleted rodents being impressively prone to post-natal inflammation and sepsis from intestinal pathogens, gnotobiotics still carry pregnancies to term and can propagate relatively depleted or replete lineages [134, 235]. Based on these observations, we can propose that mammals have intimately coevolved with microbes and now require microbes (and the diverse functions that their metagenomes encode) for normal development.

To demonstrate the necessary role of microbes in development, GF animals are introduced to microbes through conventionalization. Studies support their proof-of-principle that microbes impact development by comparing GF controls to the conventionalized animals [135, 236]. However, a few groups have compared the conventionalized animals to conventional control animals (that have always been raised with microbes), with interesting results. For example, conventionalized mice display decreased transcription of UDP-glucuronosyltransferases 1a9 and 2a3 compared to conventional mice fed a probiotic [237]. In terms of development of immune competence, one group reports that conventionalized rats harbor altered levels of intestinal intraepithelial lymphocytes compared to both their GF and conventional control counterparts [238]. They also observe that the frequency of T cells co-expressing α_4_β_7_ in the intestinal lamina propria of conventionalized mice never normalizes to the base levels of conventional controls [239]. Another group demonstrates that conventionalized mice produce decreased IFNγ, phagocytotic ability, and reactive oxygen species production in response to fungal infection compared to GF and/or conventional controls [240]. Clearly, conventionalization does not restore all aspects of atypical development caused by a GF upbringing, particularly with respect to the immune system. Several groups identify the importance of “critical developmental windows,” where reconstitution of the microbiome after said window fails to restore normal development, even partially [7–9]. In an elegant study, de Agüero *et al.* demonstrated that transient colonization of pregnant GF dams with a mutant *E. coli* improved multiple aspects of GF pup health, including intestinal group 3 innate lymphoid and F4/80 + CD11c + mononuclear cell counts, epithelial antibacterial peptide transcription levels, and microbial molecular metabolism [103]. The mutant *E. coli* was specifically engineered to not persist in the intestine, and its transience was confirmed with negative cultures of the placenta and GF status of the neonates. Despite the absence of long-term or persistent live microbial exposure, the pups demonstrated markedly improved immune health outcomes, demonstrating the essential role of the maternal microbiome in fetal immune development.

Although the living environments of GF animals are free of live microbes, they still contain detectable dead/killed microbes and microbial particles [241–243]. It is possible that these nonviable microbes and microbial components can serve as pathogen/microbe-associated molecular patterns (PAMPs/MAMPs) for immune education and other aspects of host-microbe interactions. For example, germ-free mice challenged with *Helicobacter pylori* can reduce gastric infection levels with repeated exposure to heat-killed *Lactobacillus johnsonii* [244]*.* Similarly, heat-killed *L. brevis* is associated with increasing plasma serotonin concentrations in another model [245]. It is also clearly demonstrated that viable microbes are required for proper host-microbe development [246, 247].

Observations in GF animals lead to three considerations about microbes and development.

1. GF littered animals are viable without exposure to live microbes, but they display abnormal development.
2. Even early-on conventionalization of GF animals does not fully correct this abnormal development.
3. Killed microbes and microbial components present in the living environments of GF animals may potentially contribute to immune education or other aspects of normal development.

When combined with the lessons learned from Cesarean delivery, we next consider the possibility that mammalian education for “how to form a symbiotic relationship with microbes” begins *in utero.* We will consider the possibility of a live but low-biomass microbial community within the *in utero* environment. To explore this theory, we will present the historical evidence for and against the placental microbiome and how it may contribute to the development of the offspring microbiome and long-term health.

## Does a low-abundance, low-biomass intrauterine microbiome exist?

Distinct microbial communities in the meconium among preterm and healthy-term neonates are detected and described within minutes to hours of birth, and these communities expand in the first days to weeks of life to readily show discrete body niche communities long before that same infant will alter its diet or engage in meaningful contact with the outside world [204, 248–250]. How does this happen? Traditionally, it was assumed that the healthy intrauterine environment during pregnancy (including the uterine decidua, placenta, chorion/amnion, amniotic fluid, fetus, and meconium) was sterile until birth. Only during delivery was the neonate believed to first interact with microbes from the vagina (vaginal delivery) or skin/environment (Cesarean delivery). This dogma was further supported by the negative clinical outcomes associated with prolonged and untreated/undelivered cases of chorioamnionitis (the bacterial infection of the intra-amniotic space that leads to inflammation of fetal membranes and is associated with serious morbidity and potential mortality of both the gravidae and her fetus or neonate). Chorioamnionitis is a localized infection that is most often “cured” by delivery, plus antibiotics are used to reduce the risk of post-partum endometritis. It has been argued that if bacteria were supposed to be present in the intrauterine environment, then why does chorioamnionitis occur?

As evidence continued to accumulate challenging the sterility of the intrauterine environment during pregnancy [56, 61, 158–199], so did more evidence supporting sterility [11–15, 251–272] (see Tables 1 and 2, respectively). How come research efforts seemingly performing the same or similar experiments are arriving at opposing conclusions?

**Table 1:** Historical evidence of the female intrauterine microbiome in pregnancy

Year	Endometrium or decidua	Placenta parenchyma	Chorion	Amnion	Amniotic fluid	Meconium or fetal intestine	Culture-independent Detection		Culture-dependent Detection ^**a**^		Ref.
							Method	Conclusion	Method	Conclusion	
**1926**						✓	n/a	n/a	Aerobic	38% of meconium samples are culture-positive	[158]
**1934**						✓	Histology (Löffler’s methylene blue and Gram stains)	6% of meconium samples contained recognized bacteria	Aerobic: EMB lactose, Blood, Dextrose broth, Deep iron brain	38% of meconium samples are culture-positive	[159]
**1936**						✓	Histology (Cover slip and Gram stain)	No bacteria detected	Aerobic: EMB, Blood, Brain or glucose broth. Anaerobic: Blood slants	10% of meconium samples collected within 30 min after delivery are culture-positive	[160]
**1982**			✓				n/a	n/a	Aerobic: Broth and blood agar	Aerobic bacteria have been cultured from the chorion in the absence of histologic chorioamnionitis	[161]
**1997**					✓		16S rRNA polymerase chain reaction (PCR) of the V6-8 HVRs	16S rRNA is detected in 94% of culture-positive and 36% of culture-negative amniotic fluid from cases of preterm labor with intact membranes	Traditional clinical culture	20% of amniotic fluid samples are culture-positive and 80% are culture-negative	[162]
**2004**					✓		16S rRNA sequencing of the V6-8 HVRs using Sequenase or Cycle Sequencing	16S rRNA identified in culture-negative amniotic fluid is mapped to *Leptotrichia sanguinegens, Fusobacterium nucleatum, Ureaplasma urealyticum*	Traditional clinical culture	20% of amniotic fluid samples are culture-positive and 80% are culture-negative	[163]
**2007**					✓		*Porphyromonas gingivalis* -specific PCR and 16S rRNA PCR of V5-8 HVRs	*P. gingivalis* DNA is detected in 30% of amniotic fluid from cases of threatened preterm labor	Anaerobic: non-selective Columbia blood	All amniotic fluid samples are culture-negative for *P. gingivalis*	[164]
**2007**		✓	✓	✓	✓		Gram stain of amniotic fluid samples	10% of culture-negative amniotic fluid samples yield a positive Gram stain	Traditional clinical culture	Diverse microbes are recovered from term placentas without histological or clinical evidence of inflammation	[165]
**2008**		✓	✓				16S rRNA PCR of the V3-5 HVRs, histology (Gram stain), and gas-liquid chromatography of glucose fermentation products	There is a PCR inhibitor in the placenta. At higher concentrations of placental tissue, 16S rRNA PCR products are no longer detected from known culture-positive placental samples	Aerobic: Chocolate, TSA blood. Anaerobic: A-7, Brucella blood	51% of placental samples from Cesarean-delivered and 75% from vaginally delivered preterm births are culture-positive	[166]
**2008**					✓		16S rRNA end-point PCR (V1-V4 and V3-5 HVRs) and 16S rRNA real-time PCR (V1-2 and V3-5 HVRs). Plus 18S fungal targets	Microbial rRNA is detected from culture-negative amniotic fluid of preterm labor with intact membranes	Aerobic and anaerobic	9.6% of amniotic fluid samples are culture-positive	[167]
**2008**						✓	PCR of genetically labeled *Enterococcus fecium* HA1 (that was orally inoculated into pregnant mice)	Meconium from term murine pups contains the genetic label of the *E. fecium* fed to their dams during pregnancy	Aerobic: BHI, VRB, CNA. Anaerobic: Wilkins-Chalgren, MRS	*Enterococcus* and *Staphylococcus* are cultured from meconium	[168]
**2009**		✓					*Bifidobacterium* and *Lactobacillus* -specific PCR	*Bifidobacterium* and *Lactobacillus spp.* DNA is detected in 91–97% of placental tissue	Anaerobic: Blood liver, LB	All samples are culture-negative for *Bifidobacterium spp.* and *Lactobacillus spp.*	[169]
**2010**		✓					16S rRNA PCR (primers A17F and 1512R)	After human oral bacteria are injected into murine tail vein, the 16S rRNA is detected in the murine placenta	n/a	n/a	[170]
**2010**					✓		16S rRNA end-point PCR (V1-V4 and V3-5 HVRs) and 16S rRNA real-time PCR (V1-2 and V3-5 HVRs). Plus 18S fungal targets	16S rRNA is detected from culture-negative amniotic fluid in preterm pre-labor rupture of membranes	Aerobic and anaerobic	34% of amniotic fluid samples were culture-positive	[171]
**2013**	✓						Histology (H&E, Gram, Hema 3 modified Geimsa, Brown-Hopps modified Gram stains)	Intracellular bacteria are detected by histology in the non-inflamed maternal basal plate of the placenta	n/a	n/a	[172]
**2013**						✓	16S rRNA PCR of the V6-8 HVRs analyzed by Denaturing Gradient Gel Electrophoresis and 16S rRNA Human Intestinal Tract Chip analysis of the V1-6 HVRs	Meconium microbiome is characterized by abundant Firmicutes and Proteobacteria, and is lower diversity than 3-week old infant stool	Aerobic: MRS, MacConkey, BP, SDC, BHI, CNA. Anaerobic: WC, MRS with L-cysteine	78% of meconium samples are culture-positive	[173]
**2014**		✓					16S rRNA pyrosequencing of the V1-3 HVRs with comparative WGS metagenomic sequencing	Distinct placental microbiome composed of nonpathogenic commensals, most similar to the human oral microbiome	n/a	n/a	[56]
**2014**			✓	✓			16S rRNA pyrosequencing of the V1-2 and V5-6 HVRs	Common placental genera regardless of delivery mode	n/a	n/a	[174]
**2014**	✓						Histology (Brown-Hopps modified Gram stain)	Bacteria are detected within extravillous trophoblasts of the placental basal plate	Inoculation of the placental basal plate *ex vivo*	Inoculated bacteria are detected within extravillous trophoblasts of the placental basal plate	[175]
**2015**		✓					16S rRNA sequencing of the V3-4 HVRs using Illumina MiSeq	Lower diversity and lower *Lactobacillus* percentage in placenta associated with low birthweight versus full term	n/a	n/a	[176]
**2015**		✓					16S rRNA pyrosequencing of the V1-3 HVRs with comparative WGS metagenomic sequencing	Distinct placental microbiome between women with and without excess gestational weight gain	n/a	n/a	[61]
**2016**		✓			✓	✓	16S rRNA pyrosequencing of the V1-3 HVRs with comparative quantitative PCR and denaturing gradient gel electrophoresis	Placental and amniotic fluid microbiomes both characterized by low richness, low diversity, and high Proteobacteria, which suggests microbial transfer at the fetal-maternal interface	Anaerobic: Gifu, LB	50% of taxa identified by culture-independent methods were recovered through culture	[177]
**2016**			✓				WGS metagenomic sequencing	Distinct placental microbiome in spontaneous preterm birth that further differentiates by severity of chorioamnionitis	Traditional clinical culture for *Ureaplasma* and *Mycoplasma*	Some chorioamnionitis-positive patients were negative for *Ureaplasma.* Some chorioamnionitis-negative patients were positive for *Ureaplasma*	[178]
**2016**		✓					16S rRNA sequencing of the V3-4 HVRs using Illumina MiSeq and TaqMan gene expression using RT-PCR	Distinct placental microbiome associated with gestational diabetes	n/a	n/a	[179]
**2017**		✓	✓				16S rRNA sequencing of the V6-8 HVRs using Illumina MiSeq	Fetal side of placenta harbors microbiome resembling the gravidae’s oral (not fecal) microbiome	n/a	n/a	[180]
**2017**		✓	✓	✓			16S rRNA sequencing of the V5-7 HVRs using Illumina MiSeq with comparative qPCR	Only specific bacteria found in placental tissue is associated with chorioamnionitis and low-birthweight neonates	n/a	n/a	[181]
**2017**		✓					16S rRNA sequencing of the V3-4 HVRs using Illumina MiSeq	Distinct placental microbiome associated with fetal macrosomia	n/a	n/a	[182]
**2017**	✓	✓	✓	✓			16S rRNA sequencing of all V1-9 HVRs using Illumina MiSeq with comparative qPCR (V4 region and *Ralstonia insidiosa*)	Composition of the placental microbiome differs between maternal, fetal-maternal, and fetal spaces, independent of delivery mode	n/a	n/a	[183]
**2018**		✓			✓		16S rRNA pyrosequencing of the V4 HVR	Amniotic fluid and placenta contain low-diversity microbiomes dominated by Enterobacteriaceae phylotype	Anaerobic: BHI, Columbia blood	0% of amniotic fluid samples and 20% of placenta samples are culture-positive	[184]
**2018**	✓	✓					16S rRNA sequencing of the V3-4 HVR using Illumina MiSeq with comparative qPCR	16S rRNA is detected in 49% of placental samples, with equal richness and diversity between HPV-positive and negative groups	n/a	n/a	[185]
**2019**	✓	✓	✓	✓			16S rRNA *ISH* with comparative 16S rRNA sequencing of the V4 HVR using Illumina MiSeq and histology (H&E, Warthin-Starry, and Gram stains)	Detection of low-abundance bacterial RNA in the placental villi and chorion	Aerobic: Blood, MacConkey, Chocolate, A-7. Anaerobic: Brucella, PEA, K-V	No detectable growth	[186]
**2019**					✓	✓	16S rRNA sequencing using PacBio SMRT cell	100% of meconium and 84% of amniotic fluid samples contained bacterial 16S rRNA	n/a	n/a	[187]
**2019**		✓					GFP plasmid DNA PCR and immunohistochemical detection of fluorescent-tagged *Staphylococcus aureus*	Fluorescent-tagged *S. aureus* injected into the maternal bloodstream is recovered in the placenta	BHI, TSA	No detectable growth	[188]
**2019**	✓						16S rRNA sequencing of the V5-6 HVRs using Illumina MiSeq	Bacterial DNA detected in endometrial biopsies following elective Cesarean delivery	n/a	n/a	[189]
**2019**		✓	✓	✓	✓	✓	16S rRNA sequencing of the V4-5 HVRs using Illumina MiSeq	Bacterial DNA is detected in meconium, placenta, and fetal membranes independent of delivery method	n/a	n/a	[190]
**2019**	✓	✓		✓		✓	16S rRNA *ISH* with comparative 16S rRNA sequencing of the V4 HVR using Illumina MiSeq	Bacterial DNA present in meconium partially aligns to DNA found in the *in utero* environment	Aerobic and Anaerobic: BHI, MRS, Chocolate, MacConkey	Early gestational samples are more likely to be culture-positive in murine fetus	[191]
**2019**		✓					16S rRNA sequencing of the V3-4 HVR using Illumina MiSeq with comparative qPCR	Neonatal oral microbiota most resembles the 16S rRNA profile of the placenta	n/a	n/a	[192]
**2020**		✓				✓	n/a	n/a	Aerobic and Anerobic: Blood	86.5% of meconium and 57% of placental samples are culture-positive	[193]
**2020**			✓	✓			16S rRNA sequencing of the V4 HVR using Illumina MiSeq with comparative qPCR	Bacterial DNA profiles of placental samples without histological evidence of chorioamnionitis are distinctly different than negative controls	n/a	n/a	[194]
**2020**	✓	✓	✓				*ISH* and qPCR of the 16S rRNA inter-HVR segment between the V2-3 HVRs and Fusobacterium-specific 16S segment	Preeclampsia-exposed placenta has increased total bacterial and Fusobacterium DNA load	n/a	n/a	[195]
**2020**						✓	16S rRNA sequencing of the V4 HVR using Illumina MiSeq with comparative V6 HVR qPCR, eubacterial fluorescence ISH, and scanning electron microscopy	Sparse but viable bacteria are visualized in human meconium and detected by 16S rRNA signal	Aerobic: *Micrococcus*-specific isolation in BHI	Guided by molecular identification, viable *Micrococcus spp.* isolates were cultured from meconium	[196]
**2021**		✓					16S rRNA sequencing of the V4 HVR using Illumina MiSeq following 16S Specific Enrichment	Enrichment preceding DNA sequencing allows for resolution between placental microbes and air swab controls	n/a	n/a	[197]
**2021**						✓	WGS metagenomic and metatranscriptomic sequencing using Illumina HiSeq	A low-diversity, low-biomass microbiome was identified in the prenatal fetal gut of lambs	n/a	n/a	[198]
**2021**	✓	✓	✓	✓		✓	16S rRNA sequencing of the V4-5 HVRs from nested PCR using Illumina HiSeq, 16S RNA-ISH, and scanning electron microscopy	Few but consistently present microbes are detected in healthy human fetuses in the 2nd trimester. Bacterial cocci-like shapes are localized along mucin-like structures of the fetal ileum.	Aerobic and Anaerobic: pre-reduced anaerobic blood- supplemented basal media	Tissue from 75% of fetuses produced reproducible colonies with heterogenous distribution suggesting true signal from specific organs.	[199]

^a^ Culture Media Abbreviations: *A-7* Shepard’s Differential Agar Base, *BHI* Brain Heart Infusion, *BP* Baird Parker, *CNA* Columbia Colistin-Nalidixic Acid, *EMB* Eosin Methylene Blue, *K-V* Kanamycin-Vancomycin, *LB* Luria-Bertani, *MRS* De Man, Rogosa, and Sharpe, *PEA* Phenylethyl Alcohol Agar, *PPLO* PleuroPneumonia-Like Organism, *PRAS* Pre-Reduced Anaerobically Sterilized, *PYG* Peptone Yeast Extract Glucose, *SDC* Sabouraud Dextrose Agar with Chloramphenicol, *TSA* Tryptic Soy Agar, *VRB* Violet Red Bile, *WC* Wilkins-Chalgren

**Table 2:** Historical evidence against the female intrauterine microbiome

Year	Endometrium or decidua	Placenta parenchyma	Chorion	Amnion	Amniotic fluid	Meconium or fetal intestine	Culture-independent detection		Culture-dependent detection ^**a**^		Ref.
							Method	Conclusion	Method	Conclusion	
**1958**	✓						n/a	n/a	Aerobic and anaerobic; Blood and Chocolate	1/10 patients yielded positive endometrial culture, suggests endometrium is usually sterile	[251]
**1962**					✓		n/a	n/a	Culture, unspecified	Occasional diphtheroid colonies in 2/44 abdominal punctures, suggests contamination not intrauterine infection	[252]
**1966**	✓						n/a	n/a	Aerobic: serum broth, PPLO, cooked meat, thiol, blood. Anaerobic: chocolate, thioglycolate, Casman’s	Positive endometrial culture decreases with time following intrauterine device insertion	[253]
**1967**	✓						n/a	n/a	Aerobic: blood, MacConkey. Anaerobic: blood, thioglycolate	Pathogens were cultured in 6% of endometrial samples, suggests uterine cavity is usually sterile	[271]
**1969**					✓		n/a	n/a	Aerobic: blood, desoxycholate, chocolate. Anaerobic (with H2): blood H2	44/50 negative cultures, 2/50 grew *Staphylococcus albus*. 4/50 grew *Mycoplasma hominis*. Trauma of vaginal exams introduces microbiota to enter uterus	[254]
**1970**					✓		Histology (Gram)	Unspecified	Aerobic and anaerobic culture in blood, MacConkey	1/39 positive pre-labor amniotic fluid culture. 8/78 positive mid-labor amniotic fluid cultures	[255]
**1971**					✓		Histology (Gram)	Unspecified	Aerobic and anaerobic culture	11/140 positive mid-labor amniotic fluid cultures. No maternal or fetal morbidity supports that amniotic fluid has antibacterial activity	[256]
**1976**					✓		n/a	n/a	Aerobic: TSA, blood. Anaerobic: PRAS chopped-meat glucose, blood	Postive amniotic culture in cases of obvious intraurterine infection. Amniotic fluid removed from the intact amniotic cavity appears to be sterile.	[257]
**1984**		✓		✓			Histology (Gram)	6/46 uncomplicated vaginal deliveries was positive for Gram-positive rods	Aerobic: blood, chocolate, MacConkey, thioglycolate. Anerobic: blood, KV	1/46 uncomplicated vaginal deliveries was positive (Group B Strep)	[258]
**1987**	✓						n/a	n/a	Aerobic: SP-4, 10-B, Thayer-Martin. Murine and primate tissue culture. Anaerobic: BHI	All cultures were negative for tested microbes suggesting the nonpregnant endometrium is sterile.	[259]
**2015**					✓		PCR coupled with electrospray ionization mass spectrometry	Microbes were identified in 29/231 amniotic fluid samples without the presence of intra-amniotic inflammation	Aerobic and anaerobic culture	5/231 positive amniotic fluid samples without intra-amniotic inflammation. 24/231 negative AF samples with intra-amniotic inflammation.	[260]
**2016**	✓	✓					16S rRNA sequencing of the V1-2 HVRs using Illumina MiSeq	Could not distinguish placental samples from contamination controls	n/a	n/a	[261]
**2018**	✓	✓					16S rRNA sequencing of the V1-2 HVRs using Illumina HiSeq	Could not detect placenta microbiome in preterm or term births. DNA detection limited to contamination with vaginal fluids or contaminants shared with negative controls	n/a	n/a	[262]
**2018**		✓					16S rRNA sequencing of the V5-7 HVRs using Illumina MiSeq	No evidence of reproducible preterm placental microbiome. Differences depend on placental delivery abdominally or vaginally (thus, delivery contamination)	n/a	n/a	[263]
**2018**					✓		16S rRNA sequencing of the V3-4 HVRs was quantified using digital droplet PCR then sequenced using Illumina MiSeq	No differences seen between membrane-intact and negative control amniotic fluids. Ruptured membranes was associated with higher bacterial DNA levels	Anaerobic and aerobic: BHI	No bacteria detected from membrane-intact or negative control amniotic fluids. Bacteria were detected in ruptured membrane samples	[264]
**2018**					✓		16S rRNA sequencing of the V4 HVR using Illumina MiSeq	Bacterial microbiota of amniotic fluid was indistinguishable from buffer extraction negative controls	n/a	n/a	[265]
**2018**		✓					18S rRNA sequencing of the V9 HVR using Illumina MiSeq	Unable to detect eukaryotic pathogens in placental biopsies from adverse pregnancy outcomes or healthy controls	n/a	n/a	[266]
**2019**		✓					16S rRNA sequencing of the V1-2 HVRs using Illumina HiSeq	Major source of bacterial DNA was contamination from lab reagents and equipment. Placental tissue samples become contaminated during labor and delivery. Only bacteria found before labor was *S. agalactiae*	n/a	n/a	[273]
**2019**	✓	✓	✓	✓			16S rRNA sequencing of the V4 HVR using nested PCR followed by Illumina MiSeq with comparative qPCR. WGS metagenomic sequencing using Illumina HiSeq	Could not detect microbes in Cesarean-delivered term placenta	Anaerobic and aerobic: TSA, chocolate, MacConkey	28/29 placental cultures were negative. 1/29 cultures yielded aerobic *Bacillus circulans, Bacillus pumilus,* and *Brevibacterium casei*	[267]
**2020**					✓		PCR coupled with electrospray ionization mass spectrometry. Cell-free DNA sequencing by Illumina NextSeq 500 or HiSeq. Low-biomass background correction. Histology (Gram)	Reports reduced false-positive rate, suggesting the amniotic fluid is sterile in normal pregnancy	Traditional clinical culture including genital mycoplasma	10/40 cultures were positive, all with clinical chorioamnionitis	[14]
**2020**	✓	✓	✓	✓			16S rRNA gene PCR amplification and visualization of the V2 and V4 HVRs. TaqMan qPCR of the 16S rRNA V3-5 HVRs and Erythromycin resistance methylase B gene. Histology (Gram, anti-lipoteichoic acid, anti-lipopolysaccharide). Scanning electron microscopy	No bacterial amplicons were identified (human and mouse)	TSA, chocolate, MacConkey, SDC, thioglycolate	None of the placental or environmental controls were positive for bacterial growth	[13]
**2020**		✓				✓	16S rRNA sequencing of the V4 HVR by Illumina MiSeq with quantification by 16S rRNA amplification of the V1-2 HVRs by qPCR	Bacterial loads from murine placenta and fetal tissues did not exceed those of background technical controls. Samples from maternal sites did exceed controls	TSA, chocolate, MacConkey, SP4 with urea and arginine (for Mycoplasma and Ureaplasma)	9/165 placental cultures were positive (5/9 from single mouse). 1/165 fetal intestine cultures grew *Staphylococcus hominis*	[12]
**2021**		✓			✓		16S rRNA sequencing of the V6-8 HVRs by Illumina MiSeq with comparative qPCR	Amniotic fluid and placenta have indistinguishable bacterial DNA from negative controls. No differences between elective Cesarean delivery and vaginal birth. Placenta has anti-microbial activity	Aerobic: LB. Anaerobic: *Neisseria gonorrhoeae* targeted culture	Cesarean delivery: 87/152 negative placental cultures, freq. human skin-associated genera. Vaginal delivery: 21/78 negative placental cultures, freq. human vaginal-associated genera	[15]
**2021**						✓	16S rRNA sequencing of the V3-4 HVRs by Illumina MiSeq	Neonatal rectal swabs could not be distinguished from negative controls	Anaerobic and Aerobic: Columbia, Chocolate, Schaedler, MacConkey, Sabouraud	3/20 meconium samples were positive for anaerobic and aerobic culture. 5/20 samples were positive for only one condition	[272]

^a^ Culture Media Abbreviations: *A-7* Shepard’s Differential Agar Base, *BHI* Brain Heart Infusion, *BP* Baird Parker, *CNA* Columbia Colistin-Nalidixic Acid, *EMB* Eosin Methylene Blue, *K-V* Kanamycin-Vancomycin, *LB* Luria-Bertani, *MRS* De Man, Rogosa, and Sharpe, *PEA* Phenylethyl Alcohol Agar, *PPLO* PleuroPneumonia-Like Organism, *PRAS* Pre-Reduced Anaerobically Sterilized, *PYG* Peptone Yeast Extract Glucose, *SDC* Sabouraud Dextrose Agar with Chloramphenicol, *TSA* Tryptic Soy Agar, *VRB* Violet Red Bile, *WC* Wilkins-Chalgren

The search for the intrauterine microbiome has proceeded through both culture-independent and culture-dependent methods. Culture-independent methods used to identify microbial presence within the intrauterine space include histology (most commonly but not exclusively Gram staining), immunohistology, polymerase chain reaction (PCR), 16S rRNA sequencing of the hypervariable regions (HVRs), WGS sequencing, liquid or gas chromatography, rRNA *in situ* hybridization, and scanning electron microscopy. Of these, the most popular and common method is 16S rRNA sequencing. In order to perform 16S rRNA sequencing, it is necessary to overcome a series of challenges: (1) obtain the intrauterine sample aseptically, (2) perform DNA extraction, (3) amplify the target HVR(s), (4) perform high-fidelity sequencing, and (5) apply bioinformatic approaches that are biologically relevant. Therefore, the seemingly simple approach of 16S rRNA sequencing is actually an amalgamation of multiple steps that all have the potential to introduce error. We will discuss these technical challenges and other factors that are integral to the debate of the presence or absence of the intrauterine microbiome.

### Challenge 1

Environmental microbes and contamination can potentially be construed as or obscure the signal ascribed to the placental microbiome [261]. To control for this difficulty, strict sterile procedures and rules for amount and location of starting material are required, along with inclusion of environmental swabs from every possible source of contamination (maternal body sites, surgical surfaces, DNA extraction equipment, and the sequencing site). Due to the expense of sequencing, few studies include environmental controls. Yet in the studies that do, the results are still divided in support and opposition of the intrauterine microbiome [12, 197]. However, it is important to note that the presence of the same identified microbe in both an intrauterine sample and an environment/contamination control does not automatically indicate that the microbe is a contaminant. For example, DNA signatures of *Lactobacillus spp.* commonly found in the vagina (a potential source of contamination) are also found in endometrial biopsies [274], suggesting that *Lactobacillus spp.* DNA may be present from the beginning of placental development, rather than terminally introduced during labor and delivery. In the absence of concomitantly collected samples from the same subject with deep sequencing to the species and strain level, definitive interpretations of overlapping genera as indications of contamination should exercise caution.

### Challenge 2

To distinguish the low-abundance, low-biomass communities, it is essential to include extraction control samples using just the kit reagents and no input sample. DNA extraction kit and laboratory reagents contain a collection of low-abundance microbes that yield a “kit-ome,” which complicates detection of the low-abundance, low-biomass microbial signatures [208, 268, 275–279]. On the one hand, kit negative samples are distinguishable from intrauterine microbiome signatures [56, 61, 171, 178, 181, 183, 194]. On the other hand, however, kit negatives overlap with the intrauterine microbiome patterns [11, 12, 15, 262, 263, 273, 280]. The ability or inability to distinguish intrauterine samples from negative controls is the crux of the debate. Likely reasons for this discrepancy are differences in HVR target and sequencing technology, discussed next.

### Challenge 3

The selection of primers for 16S rRNA sequencing of various HVRs is particularly influential. Some primers, such as those used by Walker *et al.* to cover the V1-2 HVRs, are designed as degenerate and yield short amplicons [281]. In contrast, other primers, such as those we used to span the V1-3 HVRs, are nondegenerate and yield longer amplicons [56]. Parnell *et al.* evaluated all nine HVRs in the context of placental samples [183]: V7-8 did not amplify at all; V1, V5, and V9 were not detected in the majority of samples; V2 and V6 generated as many reads in the negative control samples as the test samples; and V4 amplified significantly more bacterial DNA in test samples than negative control samples. This study demonstrates that, even in the same samples, the use of primers targeting different 16S rRNA HVRs can lead to drastically different conclusions.

### Challenge 4

With advances in computational power come advances in computational tools and pipelines for handling large amounts of sequencing data. Bioinformatic workflows continue to be developed and enhanced to subtract artifacts, contamination, and host DNA [282–286]. The most popular of these tools is perhaps decontam [284]. Based on frequency and prevalence, decontam relies on multiple sequencing runs on the same sample and the inclusion of negative controls, but these practices are not always possible for all researchers and situations. A new tool called Squeegee was recently released that allows for the detection of potential contaminants at the species level of low-biomass, high-host samples without the inclusion of negative controls in the sample set [287]. Squeegee assumes that DNA extraction kits and laboratory environments will share similar contaminant features. Other approaches to handle the computational demand of identifying a low-biomass community include sequencing the genomes of contaminants to definitively subtract them *post hoc* [288] or computationally controlling for the low-biomass expectation [289–291].

### Challenge 5

Alternate working definitions of “microbiota” and “microbiome” lend confusion to the field. “Microbiota” refers to the community of microbes in an ecosystem (https://www.nature.com/subjects/microbiota). As discussed earlier, “microbiome” is a more complex term with varying definitions. Whereas the *Nature* definition [https://www.nature.com/subjects/microbiome] emphasizes the genomics of the community, the Whipps *et al.* definition [1] emphasizes the ecology of the community. Together, these definitions point to a common and essential feature: functionality. Genomics, or the full content of genetic material, is a measure of capacity within a community. Ecology, or the relationship between the microorganisms, is a measure of activity within the community. Both capacity and activity are key features of function. Function is independent of the biomass of the community, be it high or low. Function is independent of the longevity of colonization. In other words, “microbiome” does not necessitate high-biomass or high abundance colonization, such as is generally implied when we consider the gut microbiome. Perhaps one of the most important questions of our time is how sparse communities remain sparse and do not increase in biomass. Furthermore, why should the functional capacity of a low-abundance, low-biomass microbiome matter? Can low numbers of microbes actually impact human physiology and development? The functional importance of these microbes still needs to be experimentally determined, but we will present the seminal research supporting and refuting a sterile intrauterine environment.

In the context of the placenta, de Goffau et al*.* analyzed placenta biopsies from over 500 human neonates plus microbial spike-in positive controls and DNA extraction negative controls using 16S rRNA sequencing with V1-2 degenerate HVR primers and a subset of metagenomic sequencing [273]. This study was strengthened by multiple cohort arms that include subjects with negative pregnancy outcomes (*n* = 318, including preeclampsia, neonates that are small for gestational age, and spontaneous preterm birth) and normal control births (*n* = 219). Researchers were blinded to the cohort arms. The focus of the study was to classify the reproducibility of microbial signals versus negative controls from placental biopsy samples. The researchers concluded that they reliably detected small microbial signatures from placental biopsies, but the signals overlapped between the placental samples and the negative controls. From this, the authors conclude that the taxonomic overlap between test samples and controls was attributable to contamination from lab reagents and equipment or contamination during labor/delivery. The only microbial signature they detected prior to the onset of labor was their microbial spike-in positive control (*Streptococcus agalactiae*). The authors note that, during sample collection, the 25 mg placental tissue was washed vigorously in sterile PBS, which is not a common methodology by others in the field. The authors also report inconsistent sequencing depth and subsequent aberrant library preparation across runs, which they assign to sequencing contamination, but could be interpreted as sequencing failure.

Subsequent work by Theis *et al.* analyzed samples (*n* = 51) from murine placental and fetal tissue via culture, quantitative real-time PCR (qPCR), and 16S sequencing, along with maternal and technical controls [12]. This study employed orthogonal methodologies to query the hypothesis of *in utero* colonization. The strengths of this study include (1) the use of an animal model to surgically acquire placental and fetal tissue before the onset of labor, and (2) orthogonal use of both culture-dependent and two non-culture-dependent methodologies. The researchers reported that only 5.5% of placental culture samples yielded a bacterial colony, and the bacterial loads of placental and fetal tissue were not higher than extraction negative controls. One sample of fetal brain yielded a higher bacterial load than background technical controls, and the microbe (*Bacillus circulans*) was traced to maternal origin. This study is limited by a lack of visualization techniques (i.e., microscopy, *in situ* hybridization) nor were germ-free mice included in analysis.

To summarize the stance of those refuting the intrauterine microbiome, Fricke *et al.* [16] asserted a “forensic approach” to detect microbiomes from bacterial DNA samples is not sufficient to infer the presence of live microbes, negating the prenatal microbiota hypothesis. However, we and other teams maintain that the *functional* importance of these microbes needs to be experimentally determined. It can be argued that these microbes are functionally important because maternal exposures bear an impact on the establishment of the offspring microbiome and subsequent health, as discussed in the first portion of this review.

Two studies in particular provide direct evidence for the *functional* significance of a low-biomass intrauterine microbiome in humans [196, 199]. In both, mid-trimester (early second trimester) human fetal tissue was examined by 16S rRNA sequencing, scanning electron microscopy, culture, and *in situ* hybridization. The multifaceted use of orthogonal approaches lends increased confidence to the discovery of microbial signatures and structures within placental and fetal organs. In particular, Rackaityte et al*.* [196] demonstrated Micrococcaceae and *Lactobacillus* spp. were key constituents in the fetal meconium, and Mishra *et al.* [199] described *Staphylococcus* and *Lactobacillus* spp. dominance patterns in fetal tissues. Both groups demonstrated with scanning electron microscopy bacteria-like cocci clustered within mucin-like structures in the fetal intestinal lumen, supporting the notion that these are nascent microbes of the fetal gut. These bacteria-like structures were not observed at 10 weeks, but were visualized at 14 weeks gestation, emphasizing the role that gestational age and temporality play in microbial detection of low-biomass communities and diminishing the likelihood of contamination. Importantly for verification of functionality from these low-biomass communities, both groups demonstrated that microbial presence can prime fetal immune education *in vitro*. Rackaityte *et al.* [196] included culture-dependent methodology in their demonstration of community functionality. Micrococcaceae isolates were cultured in media supplemented with placental steroid hormones or THP1 human monocyte cells. The fetal *Micrococcus luteus* isolate outperformed two reference *Micrococcus* strains in high-progesterone and high-β-estradiol carbon-limiting conditions, suggesting fitness specific to the *in utero* environment. Additionally, *M. luteus* alone maintained the *in vitro* capacity for intracellular survival in monocytes. In *ex vivo* experiments, *M. luteus* was exposed to primary fetal cells, which induced *TLR6* and *NKFB* expression in intestinal epithelia and demonstrated tolerogenic immunomodulation in antigen presenting cells (i.e., induced granulocyte–macrophage colony-stimulating factor, granulocyte colony-stimulating factor, interleukin-10; reduced tumor necrosis factor-α). Mishra *et al.* [199] tested the hypothesis that microbial antigens contribute to diverse T cell responses in the fetus. Two microbes (*Lactobacillus* and *Staphylococcus* spp.) were chosen that were most consistently present across fetal tissues (as measured by 16S and culture). The microbes were heat-killed and incubated with dendritic cells isolated from fetal mesenteric lymph nodes, then co-cultured with T cells from the same lymph node *in vitro*. *Staphylococcus* antigen exposure resulted in high T cell memory expansion, increased total T cell count, increased CD69 (marker of memory activation), and production of tumor necrosis factor and interferon-γ as compared to *Lactobacillus* antigen and control. Memory cells would not be stimulated by spurious contaminants; therefore, these data indicate that fetal T cells displayed activated *in vitro* memory response toward antigens from *Staphylococcus* spp. and to a lesser degree *Lactobacillus* spp. that were observed *in utero*. Collectively, these two studies demonstrate the potential functional significance that a sparse, low-biomass microbiome could have on the developing fetus and potentially long-term health outcomes of the offspring. More evidence from carefully designed and well-powered studies is needed to clarify the role of live microbes in human fetal physiology and development.

In addition to these studies demonstrating functional activity within these fetal communities, both teams of investigators addressed the risk of contamination in their findings. For example, Rackaityte *et al.* [196] included the addition of a mock community to every PCR plate for amplification and stringent technical controls for both extraction and sequencing runs. Nonetheless, Rackaityte et al*.* [196] was limited by a 30% detection of microbial profiles in fetal intestine compared to controls, and microbes were detected at very low cell numbers. Additionally, a number of sequenced samples contained fewer than 1000 microbial sequence reads. When these low-sequence read number samples were included in analysis, there was an apparent batch effect in sequencing. When these low-sequence read number samples were excluded in the analysis, there was no apparent batch effect in sequencing. Mishra *et al.* [199] overcame some of the limitations of Rackaityte *et al.* [196] and were strengthened by random and well-balanced sequencing batches and inclusion of PBS controls. However, Mishra *et al.* were hindered by the lack of maternal vaginal swabs or cultures. Fortunately, the surgical procedures used in specimen attainment are performed in highly aseptic environments [169, 172]. Although the detected microbes are known members of the vaginal microbial community, they are also likely members of the endometrial and decidual microbial communities [292, 293]. Additionally, samples were washed with PBS, and thus could have potentially been contaminated during the wash step. However, control organs (liver, spleen, lymph nodes), and 10-week fetal intestinal samples were subject to an identical PBS wash and vaginal exposure and did not yield findings akin to the > 14-week fetal intestine.

Despite the work of Mishra *et al.* and Rackaityte *et al.*, we are mindful that their work was conducted on tissues arising from the mid-gestation. We remain agnostic as to whether the placenta or other intrauterine tissues retain functional microbiomes at term gestation, and whether these communities are comprised of live microbes in a detectable abundance. Based in part on the work of Mishra *et al.* and Rackaityte et al, alongside others discussed further herein, we speculate that the presumptive low-biomass intrauterine live microbial communities may be a critical first step in ontogeny and education of the offspring immune system. A sparse microbial community within the intrauterine environment may participate in progressive pruning and maintenance of pregnancy, resistance to pathogenic organisms, and immune development tolerance of the soon-to-be neonate for more fulminant colonization by commensal microbiota in post-natal life. Little is currently understood about microbial interactions at the maternal-fetal interface. It is known that macrophages and natural killer cells are present, but it is possible that the constituent placental trophoblast cells also play a role. Trophoblasts can recognize and respond to PAMPs/MAMPs and promote regulatory cytokine secretion; in this way, trophoblasts may contribute to tolerogenic education [294]. It is interesting to note that the 16S rRNA signal we detected by *in situ* hybridization was largely localized to the synctiotrophoblast [186]. Perhaps some basal level of microbial exposure in the form of live microbes is necessary *in utero* to prevent a massive immune reaction to the microbial assault that accompanies entrance to the *ex utero* realm. Even if persistently viable microbes are absent from the intrauterine environment, it is generally understood that microbial antigenic signatures participate in offspring development. What remains to be determined with future research endeavors is the effect of live microbes versus microbial products during *in utero* development, including immune tolerance.

Experimental evidence supports both viewpoints of the “sterile” vs “not so sterile” intrauterine environment. As such, there is a critical need to fund high-quality research to clarify if, what, and how there is or is not a functional role of low-abundance, low-biomass communities (like the placental and the fetal microbiome) in potentially modulating fetal development. As we have stated since 2014, we remain uncertain if indeed there is a functional consequence to these low-biomass communities and are not confident which microbial members can be considered “nascent” to the intrauterine environment. However, it is a hypothesis that deserves ongoing rigorous testing to query the role for low-biomass microbial communities in modulating the fetal immune repertoire and early immune development. Furthermore, studies aimed at understanding *why* low-biomass communities remain sparse and *how* host immunity contributes to maintaining the sparsity are both intriguing and impactful. Given the data that pregnancy exposures are known to have long-lasting influences on offspring health and predisposition to adult-onset disease, the answers to these questions are vital to meaningful advances in scientific knowledge and public health. The current scientific debate pertaining to the functional role of low-biomass communities is far from settled and deserves ongoing mechanistic-based research with an objective lens.

## Data Availability

Not applicable

## Abbreviations

BaP: Benzo[a]pyrene
DOHaD: Developmental origins of health and disease
GF: Germ-free
HVR: Hypervariable region
PAMPs/MAMPs: Pathogen/microbe-associated molecular patterns
PCR: Polymerase chain reaction
rRNA: Ribosomal RNA gene
WGS: Whole-genome shotgun
